# Supplementary material for: ALPHA: A High Throughput System for Quantifying Growth in Aquatic Plants
Source: Plant Direct. 2025 Mar 12;9(3):e70048. doi: 10.1002/pld3.70048 (PMC11897902; doi:10.1002/pld3.70048)

# Supplemental Figures

**Supplemental Figure 1. Growth curves from salinity tolerance trial for all six clones. Y-axis values are raw pixel areas from images.**


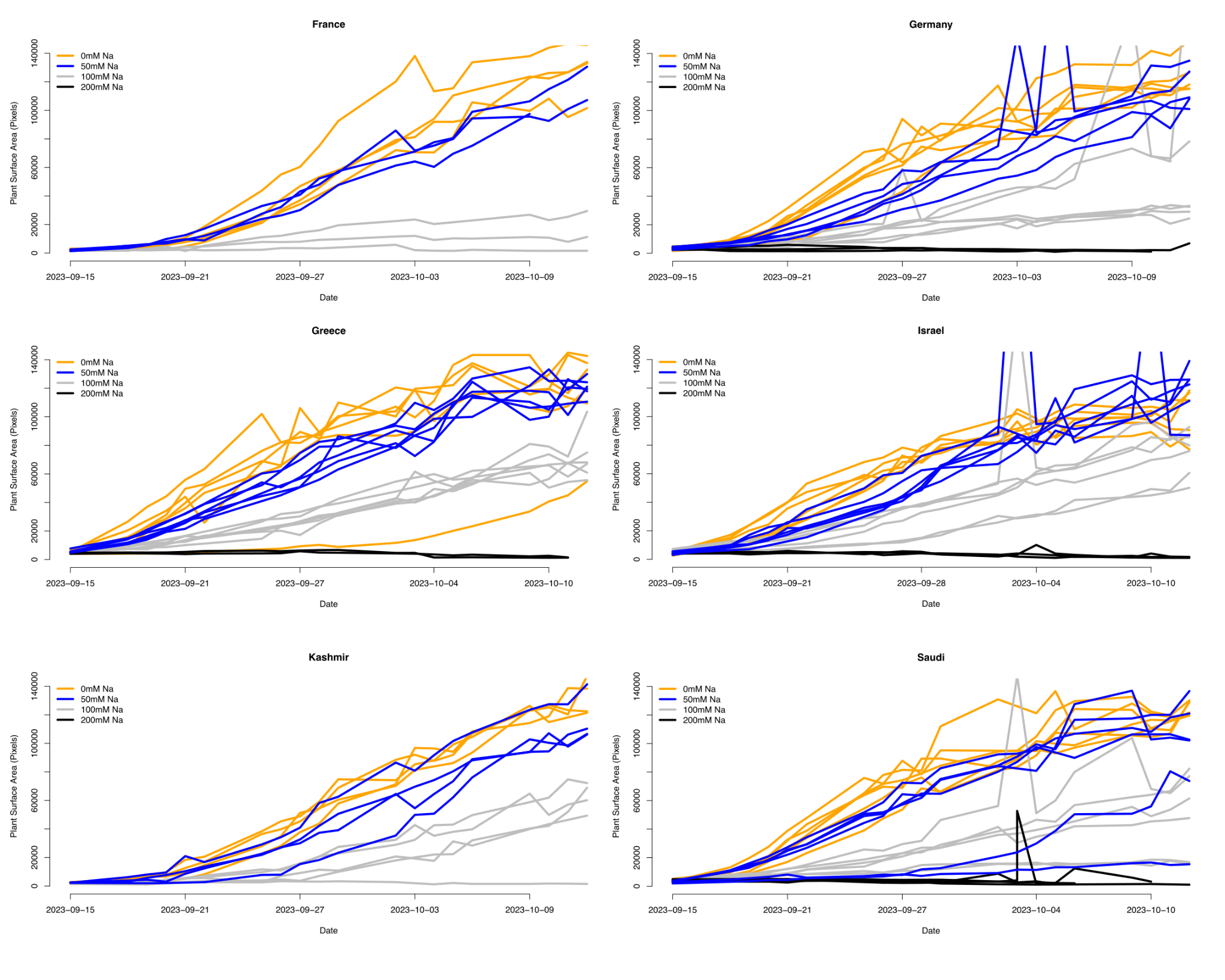


**Supplemental Figure 2. Raw images of 3 samples. Several images were excluded from the end because no growth happened during these last timepoints. The France (300mM Na) shows an example where the plant died within the first week of the experiment.**


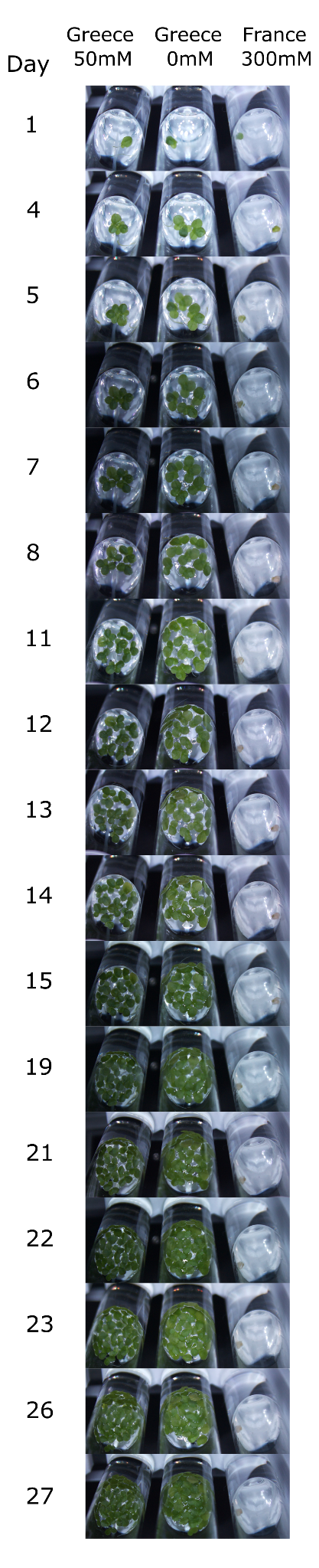

Supplement: Supplementary file 2 — Figure S1 Growth curves from salinity tolerance trial for all six clones. Y‐axis values are raw pixel areas from images. Figure S2. Raw images of three samples. Several images were excluded from the end because no growth happened during these last timepoints. The France (300 mM Na) shows an example where the plant died within the first week of the experiment. [file PLD3-9-e70048-s001.docx]
