# Supplementary material for: ALPHA: A High Throughput System for Quantifying Growth in Aquatic Plants
Source: Plant Direct. 2025 Mar 12;9(3):e70048. doi: 10.1002/pld3.70048 (PMC11897902; doi:10.1002/pld3.70048)
Supplement: Supplementary file 1 — Data S1 Peer review [file PLD3-9-e70048-s002.pdf]

Plant Direct
Open Access

ALPHA: A High Throughput System for Quantifying Growth In Aquatic Plants

Kassidy A. Robinson[a], Victoria Augoustides[c], Tanaka Madenyika[a], Ryan C. Sartor[ab*]

Decision Letter Round 1:

September 13, 2024

Dr. Ryan C Sartor
North Carolina State University
Raleigh, North Carolina

RE: ALPHA: A High Throughput System for Quantifying Growth In Aquatic Plants

Dear Dr. Sartor:

Thank you for submitting to Plant Direct. All required reviews have been returned and we have now finished our evaluation of your manuscript. In light of the reviewers' and editor's comments, further revisions are needed before the paper can be accepted for publication in Plant Direct.

Please view the editors' and reviewers' comments below and use their suggestions as a guide while you work on your revision.

When uploading the revised version of this article, please be sure to include the following:

-A word document that contains your response to the reviewers. You should respond to each reviewer comment and note the changes made to the manuscript. If you do not agree with a reviewer's comment and choose not to make a suggested revision, please explain why. Please try to provide as complete an answer as possible to each reviewer's criticisms.
-A tracked changes document with each change highlighted
- A clean version of the latest version of the manuscript

To upload your revision, please click the link below.

https://plantdirect.msubmit.net/cgi-bin/main.plex?el=A1Lr7ji6A1mnc6I3A9ftduvCp4ZYNIrTxEIQbTvM4wZ

In order to provide as timely a service as possible, we ask that your revision is resubmitted within three months after receipt of this request. If an extension is needed, please send a request, along with a brief explanation, to the editorial office at plantdirect@wiley.com .

Please note that, in addition to publishing reviewer comments, the author's responses to review comments will also be published alongside the final version of the paper. If you would not like the author's responses to be published, please contact the editorial office at plantdirect@wiley.com .

Thank you very much for giving us an opportunity to review your work. I look forward to receiving the next version.

Sincerely,

Gustavo MacIntosh

Gustavo MacIntosh

Editor, Plant Direct

-- Reviewer comments:
Reviewer #1:

This manuscript presents the development of ALPHA (Automated Lab-scale Phenotyping Apparatus), a high-throughput imaging system designed for small aquatic plants like Lemna gibba (duckweed). The manuscript is primarily a description of the mechanics of the platform, as well as the software associated with it, and a small experiment towards phenotyping a growth curve of six genotypes of L. gibba in various salt concentrations. Overall the manuscript is very well organized, presents a novel phenotyping array, builds excellent online documentation and resources, and will elevate the duckweed community. I only have some

minor comments that should be addressed.

Major comments:

-The authors acknowledge some existing efforts to phenotype duckweeds, each with pros and cons (image quality, throughput, automation, set-up cost of infrastructure, etc), and how ALPHA finds a reasonable balance between many of those barriers.

-I will not belabor the point, but the documentation on the Github page is phenomenal, including wiring diagrams, detailed instructions, and all of the code very well annotated. This is a very thoughtful way to immediately democratize the phenotyping approach here and build the duckweed community. I really commend this, and recognize the effort it takes to organize and maintain such a repository of information.

-Can individual plantlets be tracked over time for lineage tracking? In other words, in addition to just raw biomass, can we detect and track the number and location of clonal offspring?

Minor comments:

L30: change "a.k.a" to "commonly known as"
L31: not a single species, but a clade, or family would be appropriate.
L45: What is the difference between breed/develop here? Or is the better word "domesticate" perhaps since you are bringing wild species into the previous thought?
Variety and strain are used interchangeably throughout the manuscript.

Reviewer #2:

Duckweeds are a group of aquatic plant species that have become of rapid interest in recent years due to the release of genomic data and their potential to produce agricultural/synthetic products and reduce negative environmental impacts. Notably, their small size makes them very feasible to conduct whole-plant phenotyping assays in the lab. In this study, Robinson et al. describe ALPHA, an automated, throughput phenotyping platform for Lemna. The authors describe the hardware and software of ALPHA in depth, including providing a useful Github repository to make it accessible to other labs, and provide a proof-of-concept application through a salinity tolerance experiment. Overall, I believe this is a useful platform that could be beneficial to the duckweed community, which is growing, and other groups that work on other

aquatic plants. I have some comments below that I feel will improve the manuscript. These could all be considered relatively minor, but I believe are important before considering acceptance for publication:

Lines 48-53: As the authors are listing the traits, please replace the periods at the end with semi-colons.

Lines 57-67/Introduction: Parts of this introduction are written in a style that one would typically see in a grant proposal (e.g., lines 57-58: "The LIFT lab at NCSU focuses on selectively breeding Lemna gibba....."). I honestly do not have too much of an opinion on this, but the journal may require a more "standard" introduction instead of highlighting/advertising the lab's strengths like one would in a grant proposal.

I think the images in Figure 1 showing the components of the system are great, but the manuscript could really benefit if a supplemental video was added to show how the system works (as far as how the platform takes images - I do not think it's necessary to go into the data collection).

Can the authors comment whether if they had difficulties with condensation in the test tubes? I think the KimKap caps provided an immediate solution (described in Materials and Methods), but I'm curious whether it eliminated condensation over the course of multiple days to prevent difficulties of imaging.

Figure 4a: Please select colors that would be suitable for color vision-deficient readers.

Figure 4a: Why is there a lone 0mM NaCl curve at the bottom of the graph? What happened to that plant?

Figure 4a: Why did the authors only use the Greece variety here? Based on the text in the Results, I think the authors should use either the "France" or "Kashmir" variety.

Figure 4b-g: For some reason, there's a much larger gap on the X-axis of the graph between 0 - 50mM compared to 50mM-100mM, 100mM-150mM, etc.

Figure 4h: I'm assuming that here, six plants were also used for each variety. If so, it would be better to plot individual data points in the bar graphs to better show and assess the distribution of the dataset.

Could the authors provide images/timelapses from their salinity tolerance experiment and incorporate them into the manuscript?

In the Conclusion section, the authors should discuss the initial costs to set up this platform. Depending on the amount, this would allow them to also highlight that ALPHA is a cost-effective imaging platform for smaller laboratories.

ALPHA works well for Lemna and other similar sized duckweed species. But what are the authors' thoughts on using this for Wolffia (a significantly smaller duckweed species) and Landoltia (a significantly larger species)? Given that this assay uses test tubes, and the resolution of the cameras, this could be a small limitation of this platform that could be discussed in the Conclusion section.

Decision Letter Round 2:

December 22, 2024
Dr. Ryan C Sartor
North Carolina State University
Raleigh, North Carolina

MSID: 2024-01586R1
MS TITLE: ALPHA: A High Throughput System for Quantifying Growth In Aquatic Plants

Dear Dr. Ryan Sartor:

I am pleased to inform you that your manuscript "ALPHA: A High Throughput System for Quantifying Growth In Aquatic Plants" has been accepted for publication in Plant Direct.

Your article will appear online in the next available issue of Plant Direct. To ensure your article gets published as quickly as possible, please pay attention to the steps detailed below. We have found that most of the delays happen at this stage, especially at the payment stage, so please respond as quickly as possible when prompted.

License Agreement: Once your article has been accepted it will move to Production and undergo admin and file checking - you may receive an email with any queries we have at this

stage. When all required items are received by the publisher and queries resolved, the corresponding author will receive an email from Wiley's Author Services system which will ask them to log on at https://authorservices.wiley.com/bauthor and will present them with the appropriate license for completion. Your article cannot be published until both the signed license agreement and payment of the article fee have been received.

Payment of the Open Access Article Publication Fee: All articles published in Plant Direct are fully open access: immediately and freely available to read, download and share. Plant Direct charges a publication fee to cover the publication costs. The corresponding author for this manuscript should have already received a quote from the payments team (cs-openaccess@wiley.com) with the estimated article publication fee; please email cs-openaccess@wiley.com if this has not been received. The corresponding author should log on to the Wiley Author Services site, where the publication fee can be paid by credit card or an invoice. Pro Forma can also be requested. Payment of the publication charge must be received before the article will be published online.

Proofs: You will have the opportunity to look over your paper once more when you receive the author proofs for your article. The proofs will be with you in approximately two weeks. Please note that, in addition to publishing reviewer comments, the author's responses to review comments will also be published alongside the final version of the paper. If you would not like the author's responses to be published, please contact the editorial office at plantdirect@wiley.com .

Promotion of your article: You can help your research get the attention it deserves! Check out Wiley's free Promotion Guide for best-practice recommendations for promoting your work at www.wileyauthors.com/eeo/guide. And learn more about Wiley Editing Services which offers professional video, design, and writing services to create shareable video abstracts, infographics, conference posters, lay summaries, and research news stories for your research at www.wileyauthors.com/eeo/promotion.

Thank you again for your contribution to Plant Direct. If you have any questions, feel free to contact the editorial office at plantdirect@wiley.com .

Sincerely,

Gustavo MacIntosh

Plant Direct
Open Access

Editor, Plant Direct

Response to Reviewers:

Reviewer #1:

This manuscript presents the development of ALPHA (Automated Lab-scale Phenotyping Apparatus), a high-throughput imaging system designed for small aquatic plants like Lemna gibba (duckweed). The manuscript is primarily a description of the mechanics of the platform, as well as the software associated with it, and a small experiment towards phenotyping a growth curve of six genotypes of L. gibba in various salt concentrations. Overall the manuscript is very well organized, presents a novel phenotyping array, builds excellent online documentation and resources, and will elevate the duckweed community. I only have some minor comments that should be addressed.

Major comments:

-The authors acknowledge some existing efforts to phenotype duckweeds, each with pros and cons (image quality, throughput, automation, set-up cost of infrastructure, etc), and how ALPHA finds a reasonable balance between many of those barriers.

No response necessary

-I will not belabor the point, but the documentation on the Github page is phenomenal, including wiring diagrams, detailed instructions, and all of the code very well annotated. This is a very thoughtful way to immediately democratize the phenotyping approach here and build the duckweed community. I really commend this, and recognize the effort it takes to organize and maintain such a repository of information.

Thank you for the kind words. We feel that complete documentation is important.

-Can individual plantlets be tracked over time for lineage tracking? In other words, in addition to just raw biomass, can we detect and track the number and location of clonal offspring?

Great question. We have chosen not to alter the manuscript in response to this comment because the short answer is no. As the system stands, it is not capable of doing this. It may be possible to do simply with alterations to the image processing, However, the authors opinion is that this would require two changes: Less movement of culture tubes (The individual fronds will shift around when the tubes are moved to and from the imager, making tracking more difficult since colonies tend to be nearly identical); and higher time-resolution for the images (for instance an image every 10 or 30 minutes). The imaging system on its own can be used to do this in a fully automated way but this requires the culture tubes to be left within the imaging hardware for several days. The disadvantage is lower throughput (only 36 tubes at a time can be imaged) and non-controlled growth conditions (The tubes will be grown outside a growth chamber). We do feel that much of the hardware and software could be used from the ALPHA system to construct a system that would be able to meet this purpose.

Minor comments:

L30: change "a.k.a" to "commonly known as"

This has been changed in the manuscript

L31: not a single species, but a clade, or family would be appropriate.

Thank you for catching that. It has been updated to “family”

L45: What is the difference between breed/develop here? Or is the better word "domesticate" perhaps since you are bringing wild species into the previous thought?

We agree, this was poorly worded. It now reads:

"Wild plant species have the potential to contribute to this transition significantly; by leveraging natural traits and adaptations, we can domesticate wild species to produce novel crops that are better suited to meet the developing needs of human society"

Variety and strain are used interchangeably throughout the manuscript.

Thank you for pointing this out. We have changed all instances to the term "clone".

Reviewer #2:

Duckweeds are a group of aquatic plant species that have become of rapid interest in recent years due to the release of genomic data and their potential to produce agricultural/synthetic products and reduce negative environmental impacts. Notably, their small size makes them very feasible to conduct whole-plant phenotyping assays in the lab. In this study, Robinson et al. describe ALPHA, an automated, throughput phenotyping platform for Lemna. The authors describe the hardware and software of ALPHA in depth, including providing a useful Github repository to make it accessible to other labs, and provide a proof-of-concept application through a salinity tolerance experiment. Overall, I believe this is a useful platform that could be beneficial to the duckweed community, which is growing, and other groups that work on other aquatic plants. I have some comments below that I feel will improve the manuscript. These could all be considered relatively minor, but I believe are important before considering acceptance for publication:

Lines 48-53: As the authors are listing the traits, please replace the periods at the end with semi-colons.

Thank you, this has been corrected.

Lines 57-67/Introduction: Parts of this introduction are written in a style that one would typically see in a grant proposal (e.g., lines 57-58: "The LIFT lab at NCSU focuses on selectively breeding Lemna gibba....."). I honestly do not have too much of an opinion on this, but the journal may require a more "standard" introduction instead of highlighting/advertising the lab's strengths like one would in a grant proposal.

We agree that this section is not of the typical tone for peer reviewed publications. We have changed this section to read as follows:

"Nearly a century of selective breeding has made significant genetic improvements to all crop species we use today [Ahmar, 2020]. This process has been essential for the increases in yield and yield stability that we have seen over the last century. Lemnaceae will likely follow a similar path where selective breeding will play a key role in turning these wild weeds into domesticated crops and a tool for sustainable agriculture. *Lemna gibba* is the most eligible species for selective breeding due to the ease of flower induction and the ability of this species to produce viable seeds through sexual reproduction. *Lemna gibba* is the species used for the remainder of this study and will be referred to as Lemna hereafter."

I think the images in Figure 1 showing the components of the system are great, but the manuscript could really benefit if a supplemental video was added to show how the system works (as far as how the platform takes images - I do not think it's necessary to go into the data collection).

This is a great suggestion. We have added two videos (see the github page at the end of the Readme document). The first video shows how the imaging system operates and the second video shows a time-lapse sequence of duckweed growing where the images were taken with the imaging system.

Can the authors comment whether if they had difficulties with condensation in the test tubes? I think the KimKap caps provided an immediate solution (described in Materials and Methods),

but I'm curious whether it eliminated condensation over the course of multiple days to prevent difficulties of imaging.

The KimKaps work very well to prevent condensation when plants are grown near room temperature. However, we conduct many screens at elevated temperatures in an attempt to breed varieties with increased heat tolerance. Our standard breeding screen is 30°C. In these conditions, when a tube is transferred to the imager (room temperature), light condensation will form on the sides of the tubes after about 1 minute. This will tend to affect the last few images taken. We have accounted for this in the thresholding values for the image analysis software but we believe that taking advantage of more advanced image recognition methods is the best way forward. The second paragraph in the conclusion section now reads:

“While the current state of ALPHA is sufficient for use, we have identified two sources of quantification error in the system. The first is from the quantification software. Sometimes image artifacts, such as green reflections on the sides of the tubes, or imperfect images, due to condensation or unfocused cameras, can introduce errors into quantification. In the case of condensation, the use of KimKaps works well to prevent this. However, when plants are grown above room temperature, condensation will form on the tubes when brought outside the chamber for imaging. Currently no machine learning methods are being used with this system. Plant detection and quantification relies on traditional methods of thresholding on RGB values. A convolutional neural network based approach for object detection and segmentation may have advantages over the current method and may be able to alleviate the issues mentioned above. These advanced methods are currently being prototyped with ALPHA. The second source of error is biological. In practice, whenever plants are transferred into the culture tubes for phenotyping, there is a chance that they will experience shock which results in the plants dying or significant delay in the growth. This growth delay can be seen in **Figure 4a** for one of the 0 mM growth curves. This depends on both the plant variety and the experimental conditions. Many of the samples in the salt tolerance experiment died after transplanting (missing curves in **Supplemental Figure 1**). To deal with this, the system requires at least 6 biological replicates for each sample. This amount of replication also helps with the

quantification issues mentioned above. In addition, care should be taken to select healthy plants for each experiment and we recommend selecting a colony (groups of attached fronds) instead of individual fronds when starting an experiment..”

Figure 4a: Please select colors that would be suitable for color vision-deficient readers.

We have changed the colors to accommodate red-green color vision deficiency. We would like to note that 6 colors are used to represent salinity tolerance data. Within these plots, we have also made a point to label all varieties on corresponding graphs in case our choice of colors is inadequate.

Figure 4a: Why is there a lone 0mM NaCl curve at the bottom of the graph? What happened to that plant?

Please see two comments above. We have added text to the conclusion to explain this.

Figure 4a: Why did the authors only use the Greece variety here? Based on the text in the Results, I think the authors should use either the "France" or "Kashmir" variety.

The Greece variety had the highest number of replications survive and had the most consistent growth curves. See three comments above for text added to the conclusion. We have also included Supplemental Figure 1 which shows the same data for the rest of the varieties.

Figure 4b-g: For some reason, there's a much larger gap on the X-axis of the graph between 0 - 50mM compared to 50mM-100mM, 100mM-150mM, etc.

The x-axis is scaled by the natural log. The standard way to calculate EC50 is by fitting a curve to the log(concentration) vs. percent reduction in response (growth rate). As a result, it is common to plot the concentrations in log scale. See [Sree, et al.. 2015. “Natural Variance in Salt

Tolerance and Induction of Starch Accumulation in Duckweeds." *Planta* 241 (6): 1395–1404. https://doi.org/10.1007/s00425-015-2264-x.] as an example.

Figure 4h: I'm assuming that here, six plants were also used for each variety. If so, it would be better to plot individual data points in the bar graphs to better show and assess the distribution of the dataset.

Yes, replicates were used to derive these results and also the error bars. However, the final EC50 values result from curves that are fitted to all replicate data points for an individual clone. Bootstrapping is then used to estimate standard deviation. We have updated the methods section "Salinity Tolerance Analysis". It now reads:

"Next, the drc package (Ritz, 2015) was used to fit dose-response curves to the combined data of all replicates for a specific condition using a 5-factor model. The EC50 is defined as the salt concentration at which plants grow at 50% of the optimal growth rate (0mM added salt). The EC_{50} for each clone was determined using the fitted model equations and solving for 50% of the 0mM RGR value. The "boot" R package (Canty & Ripley, 2024) was used to carry out bootstrapping by randomly sampling with replacement from the replicates of each condition, re-fitting the 5-factor model and re-calculating these bootstrapped EC50s 500 times. This distribution was used to estimate the standard deviation of EC_{50}s. All data was plotted using the "ggplot2" (Wickham, 2016) and "scales" (Wickham, 2020) R packages. See the "SaltAnalysis.R" file for details and source code used."

Could the authors provide images/timelapses from their salinity tolerance experiment and incorporate them into the manuscript?

We have added Supplemental Figure 2.

In the Conclusion section, the authors should discuss the initial costs to set up this platform. Depending on the amount, this would allow them to also highlight that ALPHA is a cost-effective imaging platform for smaller laboratories.

This is a great suggestion. We have added the following:

“The material cost of the system is within the budget of most labs. Materials for one imager is about $2000. The plant growth chambers are the most expensive part but most plant labs will already have access to growth chambers. The custom racks can be printed for ~$15/rack worth of material (PLA filament) or can be acquired from a 3D printing service for ~$30/rack. An averaged size, single door growth chamber with 4 shelves can hold 40-50 of these racks and grow 720-900 individual samples at a time. When 900 tubes are utilized for an experiment, a technician can image all of these is less than 1 hour if two imagers are available. The system is designed to use reusable Pyrex® Vista 20 x150mm rimless culture tubes that cost ~$1 each with Kimkap lids that cost ~$0.40 each.”

ALPHA works well for Lemna and other similar sized duckweed species. But what are the authors' thoughts on using this for Wolffia (a significantly smaller duckweed species) and Landoltia (a significantly larger species)? Given that this assay uses test tubes, and the resolution of the cameras, this could be a small limitation of this platform that could be discussed in the Conclusion section.

We have added the following text to the conclusion section:

“The system is designed for use with medium sized Lemnaceae species such as the *Lemna* and *Landoltia* genera. We have successfully imaged smaller Lemna species (about 1/5 the size of *Lemna gibba* shown here). However we have not tested the smallest species, such as *Wolffia globosa*. Such species may require reconfiguration of the quantification software but would likely be possible to quantify with this system. Larger species in the *Spirodela* genus may be too large for the culture tubes used here. However, many of the components of the system could be repurposed for use with larger culture tubes if the goal was to phenotype larger species.”
